# Zidovudine, an anti-viral drug, resensitizes gemcitabine-resistant pancreatic cancer cells to gemcitabine by inhibition of the Akt-GSK3*β*-Snail pathway

**DOI:** 10.1038/cddis.2015.172

**Published:** 2015-06-25

**Authors:** T Namba, R Kodama, S Moritomo, T Hoshino, T Mizushima

**Affiliations:** 1 Science Research Center, Kochi University, Kochi 783-8505, Japan; 2 Graduate School of Medical and Pharmaceutical Sciences, Kumamoto University, Kumamoto 862-0973, Japan; 3 Department of Analytical Chemistry, Faculty of Pharmacy, Keio University, Tokyo 105-8512, Japan

## Abstract

Pancreatic cancer is one of the most difficult malignancies to treat owing to the rapid acquisition of resistance to chemotherapy. Gemcitabine, a first-line treatment for pancreatic cancer, prolongs patient survival by several months, and combination treatment with gemcitabine and other anti-cancer drugs in the clinic do not show any significant effects on overall survival. Thus, identification of a drug that resensitizes gemcitabine-resistant pancreatic cancer to gemcitabine and a better understanding of the molecular mechanisms of gemcitabine resistance are critical to develop new therapeutic options for pancreatic cancer. Here, we report that zidovudine resensitizes gemcitabine-resistant pancreatic cancer to gemcitabine as shown by screening a compound library, including clinical medicine, using gemcitabine-resistant cells. In analyzing the molecular mechanisms of zidovudine effects, we found that the epithelial-to-mesenchymal transition (EMT)-like phenotype and downregulation of human equilibrative nucleoside transporter 1 (hENT1) are essential for the acquisition of gemcitabine resistance, and zidovudine restored these changes. The chemical biology investigations also revealed that activation of the Akt-GSK3*β*-Snail1 pathway in resistant cells is a key signaling event for gemcitabine resistance, and zidovudine resensitized resistant cells to gemcitabine by inhibiting this activated pathway. Moreover, our *in vivo* study demonstrated that co-administration of zidovudine and gemcitabine strongly suppressed the formation of tumors by gemcitabine-resistant pancreatic cancer and prevented gemcitabine-sensitive pancreatic tumors from acquiring gemcitabine-resistant properties, inducing an EMT-like phenotype and downregulating hENT1 expression. These results suggested that co-treatment with zidovudine and gemcitabine may become a novel therapeutic strategy for pancreatic cancer by inhibiting chemoresistance-specific signaling.

Pancreatic cancer is the one of the most lethal malignancies, and it is the fourth leading cause of cancer-related deaths worldwide. Most patients receive chemotherapy because pancreatic cancer is difficult to diagnose at an early stage because of the lack of established detection methods.^1, 2^ Gemcitabine (2', 2'-difuluorodeoxycytidine) is a current standard chemotherapeutic agent for advanced disease. Gemcitabine not only suppresses tumor growth but also improves patient quality of life and prolongs survival by several months.^3, 4^ Many clinical trials have been performed to identify a treatment that can enhance the effects of gemcitabine, but only elrotinib has been found to extend median survival by an additional 14 days.^5, 6, 7, 8, 9^ Thus, a new strategy of gemcitabine combination chemotherapy is needed. One of the most important explanations for the limited effect of gemcitabine is the rapid development of gemcitabine resistance in pancreatic cancer. Thus, an understanding of the molecular mechanisms underlying gemcitabine resistance and the development of a therapeutic agent to target gemcitabine-resistant mechanisms may lead to the development of effective gemcitabine therapeutic methods for pancreatic cancer.

Human equilibrative nucleoside transporter 1 (hENT1) is a widely distributed facilitated diffusion transporter, which uptakes purine and pyrimidine nucleosides into cells.^10^ Several clinical trials have revealed that hENT1 is an important factor in gemcitabine sensitivity. Patients who have high hENT1 expression levels in their tumors have longer median overall survival times with gemcitabine treatment than patients who have low hENT1 expression levels.^11, 12, 13^ However, it is still unclear whether gemcitabine treatment affects hENT1 expression levels.

Several reports have suggested that there is a molecular relationship between gemcitabine resistance and the epithelial-to-mesenchymal transition (EMT) phenotype.^14, 15, 16^ EMT is characterized by the loss of cell–cell junctions resulting in the formation of migratory mesenchymal cells with invasive properties. Several pathways are known to induce EMT, including the transforming growth factor (TGF)-*β*, Notch, and Wnt pathways, and the Notch pathway is involved in gemcitabine-induced EMT.^15, 17^ EMT is related to cancer aggressiveness, such as metastasis and chemoresistance, in several types of cancer, including pancreatic cancer.^16, 18^ Thus, targeting EMT cells or preventing the induction of EMT may be new targets for anti-cancer therapeutic methods.

Zidovudine was developed from spongothymidine isolated from *Cryptotethia crypta*, and it is a thymidine analog and anti-viral drug used to treat HIV infection through the inhibition of reverse transcriptase.^19^ Recently, zidovudine has been shown to have anti-cancer effects against adult T-cell leukemia/lymphoma and Kaposi sarcoma.^20, 21^ However, the effects of zidovudine on pancreatic cancer and zidovudine anti-cancer targets are largely unknown.

In this study, we screened compounds that resensitize gemcitabine-resistant pancreatic cancer cells to gemcitabine-induced cell death using a chemical library, which contains ~470 compounds. Here, we report that zidovudine enhanced gemcitabine-induced cell death in highly gemcitabine-resistant pancreatic cancer cells. Analysis of the molecular mechanisms of zidovudine effects revealed that suppression of hENT1 expression and induction of an EMT-like phenotype via activation of the Akt-GSK3*β*-Snail1 pathway are essential for gemcitabine resistance. Interestingly, zidovudine inhibited these chemoresistant phenotypes by suppressing this activated pathway. Moreover, zidovudine and gemcitabine co-treatment suppressed *in vivo* tumor growth induced by gemcitabine-resistant pancreatic tumor cells, and it inhibited the acquisition of the gemcitabine-resistant phenotype in xenografted tumors induced by gemcitabine-sensitive cells. Thus, zidovudine and gemcitabine co-treatment is a new chemotherapeutic strategy for pancreatic cancer by targeting gemcitabine resistance.

## Results

### Zidovudine selectively enhances gemcitabine-induced cell death in gemcitabine-resistant pancreatic cancer cells

**Figure 1:**
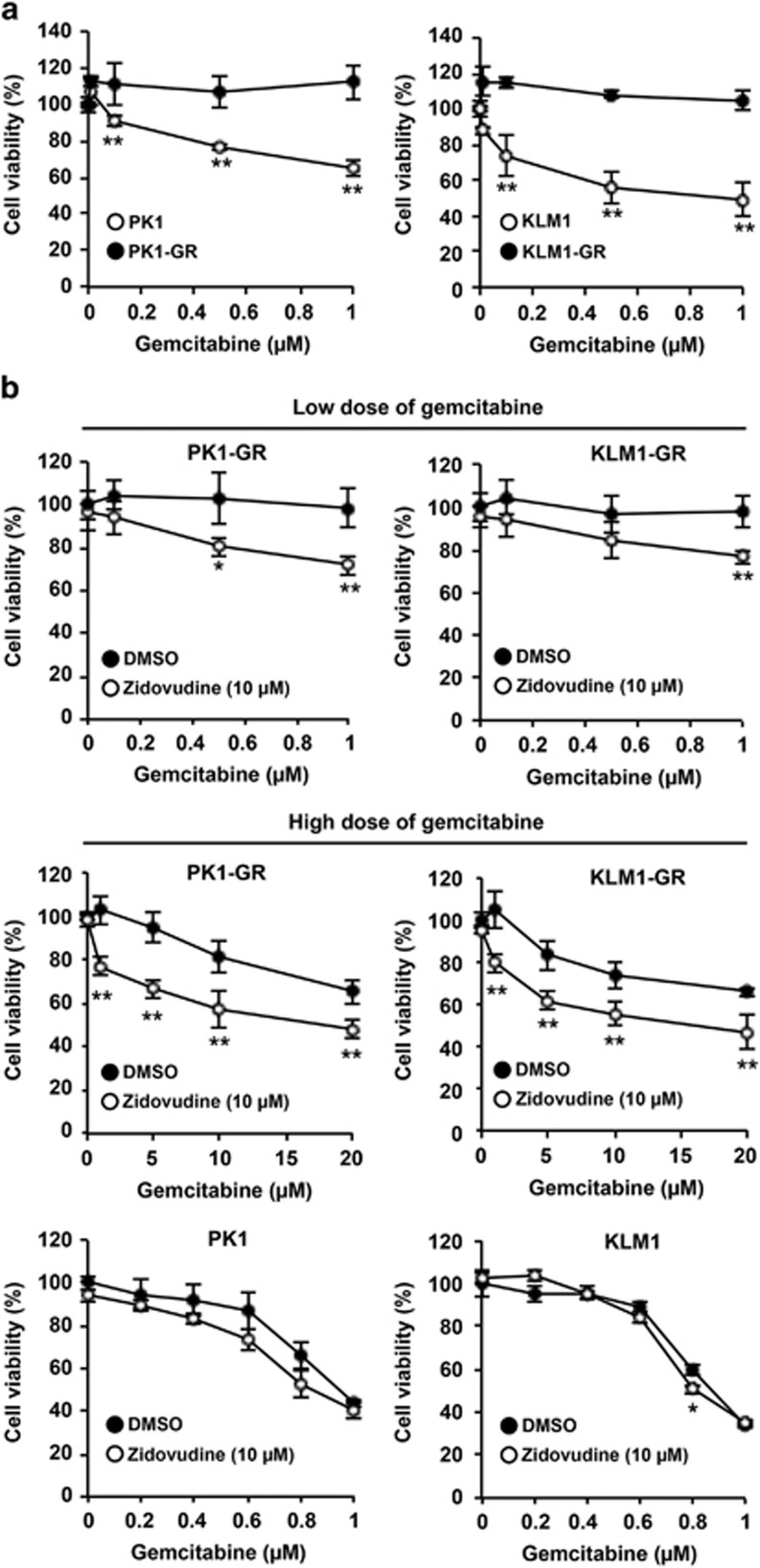
Zidovudine resensitizes gemcitabine-resistant pancreatic cancer cells to gemcitabine. (**a**) PK1-GR and KLM1-GR cells are resistant to gemcitabine-induced cell death. PK1, KLM1, PK1-GR, or KLM1-GR cells were incubated with gemcitabine at the indicated concentrations for 72 h. Cell viability was determined using an MTT assay. (**b**) Zidovudine selectively enhances gemcitabine-induced cell death in PK1-GR and KLM1-GR cells. PK1, KLM1, PK1-GR, or KLM1-GR cells were co-treated with zidovudine (10 *μ*M) and gemcitabine (at the indicated concentrations) for 72 h using the procedure described in **a**. The values shown are the mean±standard deviation (S.D.) of three different experiments measured simultaneously. The *P* value was calculated using two-way ANOVA. *P<0.05; **P<0.01 (**a** and **b**)

Gemcitabine-resistant pancreatic cancer cells were induced in PK1 (PK1-GR) and KLM1 (KLM1-GR) cells via serially increasing gemcitabine concentrations. Treatment with 1 *μ*M gemcitabine induced death in ~40% of PK1 and KLM1 cells. However, gemcitabine treatment did not induce cell death in PK1-GR and KLM1-GR cells (Figure 1a).

Using 478 compounds already in clinical use and secondary metabolites from marine microorganisms (Supplementary Table 1), we screened for compounds that resensitized the gemcitabine-resistant pancreatic cancer cells to gemcitabine. PK1-GR and KLM1-GR cells were co-treated with 1 *μ*M gemcitabine and each of the 478 compounds using a concentration that had no effect on cell viability in these cells. Several compounds, such as loperamide, promethazine, cloperastine, and zidovudine, revealed the expected effect of resensitizing the cells to gemcitabine. In these compounds, zidovudine showed a strong effect on it at low concentration (Supplementary Figure 1A) and did not induce cell death up to 50 *μ*M in PK1-GR and KLM1-GR cells (Supplementary Figure 1B). Thus, we selected zidovudine for further experiments. We confirmed that zidovudine significantly stimulated gemcitabine-induced cell death in PK1-GR and KLM1-GR cells (Figure 1b upper and middle panel), but it did not induce cell death in PK1 and KLM1 cells (Figure 1b lower panel). These results indicated that zidovudine selectively inhibits the activated signaling pathway in the gemcitabine-resistant cells.

### Zidovudine anti-viral effects are not related to resensitization to gemcitabine

**Figure 2:**
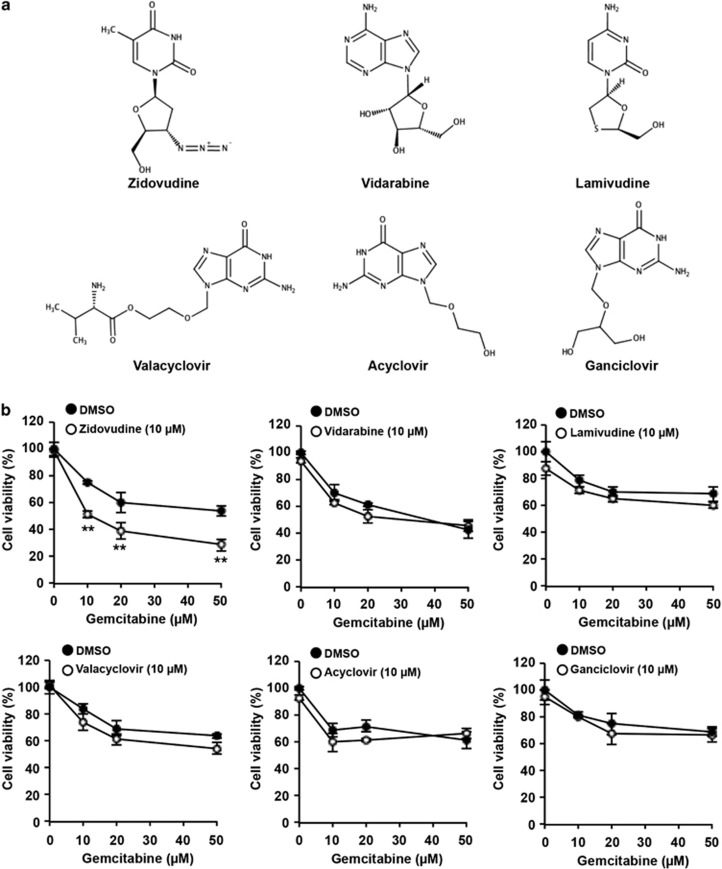
The zidovudine-enhanced effect of gemcitabine-induced cell death does not depend on anti-viral effects. (**a**) Chemical structure for indicated drug. (**b**) PK1-GR cells were co-treated with indicated drug (10 *μ*M) and gemcitabine (at the indicated concentrations) for 72 h. Cell viability was determined using an MTT assay. The values shown are the mean±S.D. of three different experiments measured simultaneously. The *P* value was calculated using two-way ANOVA. **P<0.01 (**b**)

To determine the zidovudine anti-viral effects involved in the resensitization to gemcitabine, we co-treated PK1-GR cells with gemcitabine and anti-viral drugs, including vidarabine, lamivudine, valacyclovir, acyclovir, and ganciclovir, which are nucleotide analogs that inhibit viral DNA polymerase or reverse transcriptase in PK1-GR cells (Figure 2a). A single treatment of these drugs did not significantly affect cell viability, and simultaneous treatment with gemcitabine showed no effect on gemcitabine-induced cell death (Figure 2b). Thus, the anti-viral effects of zidovudine were not involved in the stimulation of gemcitabine-induced cell death, suggesting that zidovudine has a specific target that is essential for gemcitabine resistance.

### Zidovudine upregulates hENT1 expression

**Figure 3:**
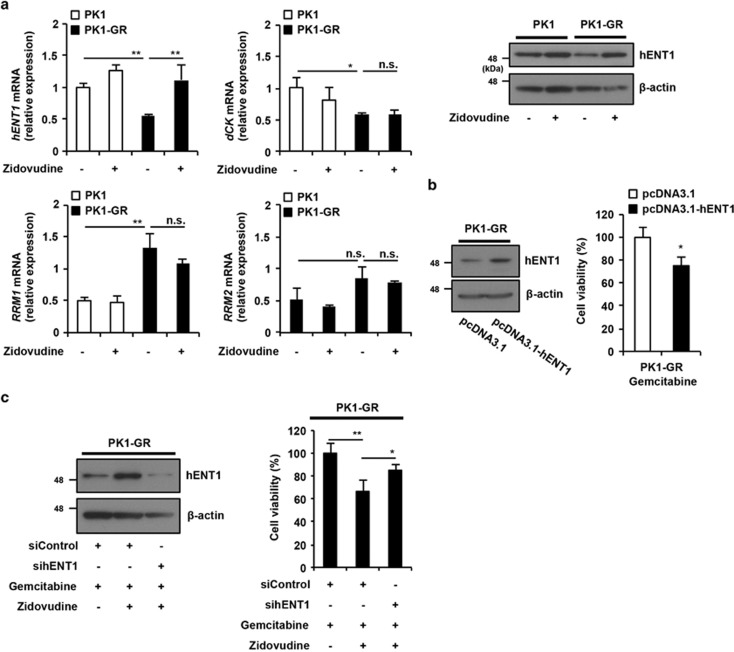
Zidovudine upregulates hENT1 expression, and this induction is involved in the zidovudine-dependent stimulation of gemcitabine-induced cell death in PK1-GR cells. (**a**) Zidovudine recovers the suppressed hENT1 mRNA and protein expression levels in PK1-GR cells. PK1 or PK1-GR cells were incubated with zidovudine (10 *μ*M) for 48 h. Left panel: Total RNA was extracted and subjected to qRT-PCR analysis using specific primer sets for *hENT1*, *dCK*, *RRM1*, *RRM2*, and *β-actin*, and the data were normalized to those of *β-actin*. Upper right panel: whole-cell lysates were subjected to immunoblotting with the indicated antibodies. The blot was stripped and reprobed. (**b**) hENT1 overexpression enhances gemcitabine-induced cell death in PK1-GR cells. hENT1 was transiently expressed in PK1-GR cells. After 24 h, these cells were treated with gemcitabine (1 *μ*M) for 48 h using the procedure described in **a** and Figure 1. (**c**) Knockdown of hENT1 expression restores survival in gemcitabine and zidovudine co-treated PK1-GR cells. PK1-GR cells were manipulated with siControl or sihENT1 for 24 h and were then treated with gemcitabine (1 *μ*M) and/or zidovudine for 72 h using the procedure described in **a** and Figure 1. The values shown are the mean±S.D. of three (**a** and **b**) or four (**c**) different experiments measured simultaneously. The *P* value was calculated using two-way ANOVA. NS, not significant; *P<0.05; **P<0.01 (**a**–**c**)

Numerous reports have suggested that hENT1 expression is a key factor in determining the anti-cancer efficiency of gemcitabine.^12, 22, 23^ Downregulation of the expression of deoxycytidine kinase (dCK), which converts gemcitabine monophosphate to its active metabolites, and the upregulation of the expression of ribonucleoside diphosphate reductase 1, 2 (RRM1, 2), which is a target molecule of gemcitabine, also suggest resistance to gemcitabine treatment.^13, 24, 25^ Therefore, we investigated whether zidovudine affects the expression levels of hENT1, dCK, RRM1, and RRM2 in gemcitabine-resistant pancreatic cancer cells. PK1-GR and KLM1-GR cells were treated with zidovudine and analyzed by real-time PCR and immunoblotting methods. The downregulation of *hENT1* and *dCK* mRNA expression and upregulation of *RRM1* mRNA expression occurred in PK1-GR cells compared with PK1 cells (Figure 3a left panel). Interestingly, zidovudine treatment upregulated the expression of *hENT1* mRNA in PK1-GR cells. The hENT1 protein expression was also upregulated by zidovudine treatment in PK1-GR and KLM1-GR cells (Figure 3a upper right panel and Supplementary Figure 2A). Next, we determined whether the zidovudine-dependent upregulation of hENT1 expression is involved in stimulating gemcitabine-induced cell death by transiently overexpressing hENT1 or knockdown of hENT1 expression by its siRNA. As expected, hENT1 overexpression stimulated gemcitabine-induced cell death in PK1- and KLM1-GR (Figure 3b and Supplementary Figure 2B), and hENT1 knockdown suppressed the co-treatment of gemcitabine- and zidovudine-induced hENT1 expression and cell death in PK1-GR (Figure 3c), suggesting that the upregulation of hENT1 expression is one of the mechanisms used by zidovudine for inducing resensitization to gemcitabine.

### Zidovudine suppresses the EMT-like phenotype

**Figure 4:**
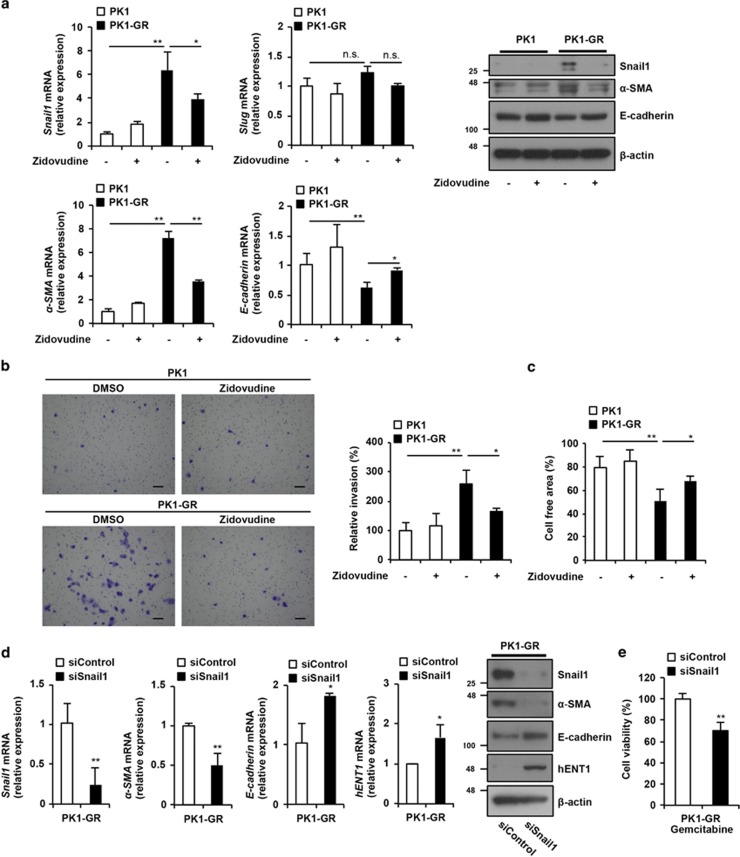
Zidovudine suppresses the EMT-like phenotype, and this suppression is involved in the zidovudine-dependent stimulation of gemcitabine-induced cell death in PK1-GR cells. (**a**) PK1 or PK1-GR cells were incubated with zidovudine (10 *μ*M) for 48 h. Left panel: Total RNA was extracted and subjected to qRT-PCR analysis using specific primer sets for *Snail1*, *Slug*, *α-SMA*, *E-cadherin*, and *β-actin*, and the data were normalized to those of *β-actin*. Upper right panel: Whole-cell lysates were subjected to immunoblotting with the indicated antibodies. The blot was cut based on the size of proteins or stripped and reprobed. (**b**) Zidovudine strongly suppresses invasion activity in PK1-GR cells. PK1 or PK1-GR cells were incubated with or without zidovudine (10 *μ*M) for 48 h, and these cells were then seeded on Transwell chamber coated with Matrigel. After 24 h, invaded cells were stained with crystal violet (scale bar, 100 *μ*m; left panel). Stained crystal violet was extracted and measured at 570 nm (right panel). (**c**) Zidovudine suppresses migration activity in PK1-GR cells. PK1 or PK1-GR cells were incubated with or without zidovudine (10 *μ*M) for 48 h, and cells were wounded. The cell-free area was measured after 18 h of incubation and expressed as relative to that before incubation. (**d**) Zidovudine-dependent suppression of Snail1 expression has an essential role in the induction of the EMT-like phenotype in PK1-GR cells. PK1-GR cells were transfected with siControl or siSnail1 for 48 h. Left panel: Total RNA was extracted and subjected to qRT-PCR analysis using the indicated specific primer sets. Right panel: Whole-cell lysates were subjected to immunoblotting with the indicated antibodies. (**e**) Suppression of Snail1 expression enhances the effect of gemcitabine-induced cell death in PK1-GR cells. PK1-GR cells transfected with siControl or siSnail1 were treated with gemcitabine (1 *μ*M) for 48 h. Cell viability was determined using an MTT assay. The values shown are the mean±S.D. of three (**a**, **b**, **d** and **e**) or four (**c**) different experiments measured simultaneously. The *P* value was calculated using two-way ANOVA. NS, not significant; *P<0.05; **P<0.01 (**a**–**e**)

To investigate whether the EMT phenotype, which has been well established as being related to gemcitabine resistance, is induced in PK1-GR and KLM1-GR cells and whether zidovudine affects the EMT phenotype, we examined the mRNA and protein expression levels of markers for epithelial cells (E-cadherin) and mesenchymal cells (Snail1, Slug, and *α*-smooth muscle action (*α*-SMA)).^17^ As shown Figure 4a and Supplementary Figure 2A, PK1-GR and KLM1-GR cells had upregulated expression levels of Snail1 and *α*-SMA (mesenchymal cell markers) as well as downregulated expression levels of E-cadherin (an epithelial cell marker). However, the expression levels of Slug did not change. In PK1-GR and KLM1-GR cells, treatment with zidovudine downregulated the expression levels of Snail1 and *α*-SMA, and it upregulated the expression level of E-cadherin. However, zidovudine treatment did not affect the expression levels of these marker proteins in PK1 and KLM1 cells. To further confirm that PK1-GR shows an EMT phenotype, we examined cell invasion and migration activity by Boyden-chamber assay using Matrigel-coated chamber and wound-healing assay.^26^ PK1-GR cells showed an increased invasion activity compared with PK1 cells, and significantly suppressed cell invasion by zidovudine treatment in PK1-GR cells (Figure 4b). Furthermore migration activity was also increased in PK1-GR cells and was suppressed by zidovudine (Figure 4c). Together, these results suggested that PK1-GR and KLM1-GR cells show an EMT-like phenotype and that zidovudine suppresses the EMT-like phenotype in these cells.

Snail1 is a transcriptional factor that mediates EMT, and overexpression of Snail1 can induce EMT in several tumor types.^27, 28, 29^ Therefore, we hypothesized that zidovudine-dependent downregulation of Snail1 expression in gemcitabine-resistant pancreatic cancer cells is one of the mechanisms used to suppress the EMT-like phenotype and resensitize cells to gemcitabine. To test this hypothesis, we demonstrated the effect of downregulated Snail1 on the EMT-like phenotype and gemcitabine-induced cell death. The downregulation of Snail1 expression using siRNA suppressed the EMT-like phenotype and induced both hENT1 mRNA and protein expression in PK1-GR and KLM1-GR cells (Figure 4d and Supplementary Figure 2C). Moreover, expression of Snail1 siRNA stimulated gemcitabine-induced cell death (Figure 4e and Supplementary Figure 2D). Overall, these results suggested that downregulation of Snail1 expression by zidovudine has a key role in zidovudine-dependent suppression of the EMT-like phenotype and resensitization to gemcitabine.

Snail1 downregulation also upregulated hENT1 expression (Figure 4d), suggesting that hENT1 expression may be involved in the induction of EMT. Thus, we investigated whether treatment with TGF-*β*, an EMT inducer, affects the expression of hENT1 in PK1 cells. Treatment with TGF-*β* induced the EMT-like phenotype and suppressed hENT1 expression in PK1 cells (Supplementary Figure 3). These results indicated that hENT1 expression is partially controlled by Snail1.

### Inhibition of the activated Akt-GSK3*β* pathway resensitizes cells to gemcitabine-induced cell death

**Figure 5:**
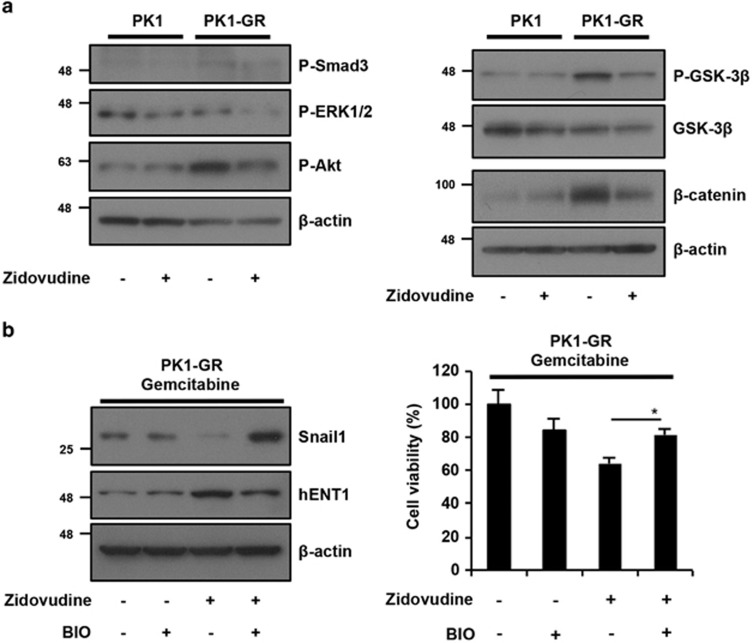
Activation of the Akt-GSK3*β* pathway is important for gemcitabine resistance. (**a**) The activated Akt-GSK3*β* pathway is suppressed by zidovudine in PK1-GR cells. PK1 or PK1-GR cells were incubated with zidovudine (10 *μ*M) for 48 h. Whole-cell lysates were subjected to immunoblotting with the indicated antibodies. The blot was cut based on the size of proteins or stripped and reprobed. (**b**) The GSK3*β* inhibitor suppresses gemcitabine and zidovudine co-treatment-induced cell death in PK1-GR cells. PK1-GR cells were treated with gemcitabine and simultaneously co-cultured with zidovudine (10 *μ*M) and/or BIO (10 *μ*M) for 48 h. Left panel: Whole-cell lysates were analyzed using the procedure described in **a**. Right panel: Cell viability was determined using an MTT assay. The values shown are the mean±S.D. of three different experiments measured simultaneously. The *P* value was calculated using two-way ANOVA. *P<0.05 (**b**)

As shown in Figure 4d,Supplementary Figures 2C and 3, Snail1 regulated hENT1 expression and the EMT-like phenotype. Thus, we investigated the molecular mechanism of zidovudine-dependent suppression of Snail1 expression as a key signal transducer for zidovudine-induced resensitization to gemcitabine-induced cell death. Treatment with zidovudine suppressed the expression level of *Snail1* mRNA by ~30% in PK1-GR cells. However, the expression level of Snail1 protein was almost completely suppressed by zidovudine (Figure 4a). Thus, we hypothesized that zidovudine suppressed Snail1 expression via regulation of transcription and protein stability. Activation of Smad3 and ERK1/2 (phosphorylated form; P-Smad3 (Ser 423/425) and P-ERK1/2 (Thr 202/Tyr 204)) signaling mainly stimulates *Snail1* mRNA transcription, and active GSK3*β* (dephosphorylating form) stimulates Snail1 protein degradation.^30, 31^ Phosphorylated GSK3*β* (Ser 9; P-GSK3*β*) induced by phosphorylated Akt (Ser 473) (P-Akt) is inactive.^30^ Therefore, we tested whether the phosphorylation of Smad3, ERK1/2, Akt, and GSK3*β* is induced in PK1-GR cells and whether treatment with zidovudine suppresses the phosphorylation of these kinases. The expression levels of P-Akt and P-GSK3*β* were increased in PK1-GR cells compared with PK1 cells, and the phosphorylation levels were suppressed by zidovudine (Figure 5a and Supplementary Figure 2E). The expression levels of P-Smad3 and P-ERK1/2 did not change, suggesting that *Snail1* mRNA is regulated by other factors. Active GSK3*β* stimulates *β*-catenin protein degradation. Similar to Snail1 expression, *β*-catenin expression was increased in PK1-GR cells, and this induction was suppressed by zidovudine (Figure 5a right panel). These results indicated that Akt activation and inhibition of GSK3*β* activity upregulated Snail1 expression via increasing protein stability, and this signal transduction stimulated gemcitabine resistance. Furthermore, zidovudine-dependent inhibition of the Akt-GSK3*β* signaling pathway induces Snail1 degradation and resensitization to gemcitabine.

To further confirm our hypothesis, we tested whether the GSK3*β* inhibitor BIO could prevent the resensitization to gemcitabine-induced cell death by zidovudine. The expression levels of Snail1 and hENT1 decreased and increased, respectively, in PK1-GR and KLM1-GR cells with zidovudine treatment, and these expression changes were suppressed by simultaneous treatment with BIO (left panel of Figure 5b and Supplementary Figure 2F). The cell death induced by the co-treatment of PK1-GR and KLM1-GR cells with zidovudine and gemcitabine was suppressed by simultaneous treatment with BIO, the GSK3*β* inhibitor (right panel of Figure 5b and Supplementary Figure 2F). Together, these results indicated that activation of GSK3*β* is essential for zidovudine-dependent resensitization to gemcitabine-induced cell death in gemcitabine-resistant pancreatic cancer cells.

### Zidovudine limits the growth of gemcitabine-resistant pancreatic tumors and inhibits the acquisition of gemcitabine resistance *in vivo*

**Figure 6:**
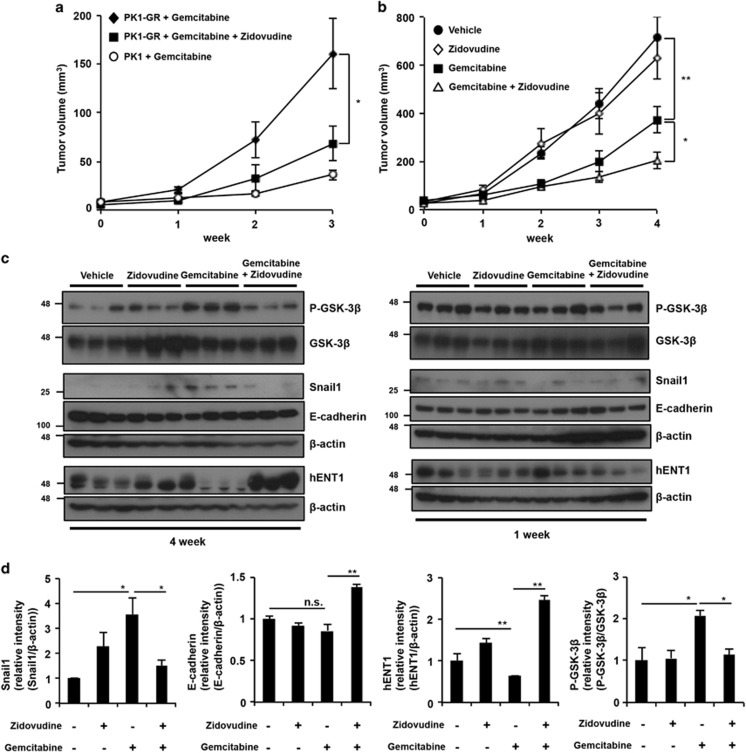
Co-treatment with zidovudine and gemcitabine suppresses the growth of gemcitabine-resistant pancreatic cancer cells and inhibits the gemcitabine-resistant phenotype *in vivo*. (**a**) Co-administration of gemcitabine and zidovudine suppresses the growth of gemcitabine-resistant tumors in nude mice. PK1-GR or PK1 cells were used for xenograft transplantation in nude mice. Two days after injecting the cells, gemcitabine (10 mg/kg) or DMSO was intraperitoneally administered once every 2 days, and zidovudine (100 mg/kg) or 1% methylcellulose was administered orally once a day (vehicle, administration of DMSO and 1% methylcellulose). Tumor volumes were measured on the indicated days. (**b** and **c**) Zidovudine inhibits the gemcitabine-resistant phenotype in pancreatic tumors. PK1 cells were used for xenograft transplantation in nude mice. Seven days after injecting the cells, gemcitabine (10 mg/kg) or DMSO was intraperitoneally administered once every 3 days, and zidovudine (100 mg/kg) or 1% methylcellulose was administered orally once a day. (**b**) Tumor volume was measured on the indicated days. (**c**) Xenograft tumors were removed and subjected to immunoblot. Each immunoblot band showed different xenograft tumors. (**d**) Administration of zidovudine suppresses gemcitabine-induced activation of the GSK3*β*-Snail1 pathway and inhibition of hENT1 expression. The intensities of the P-GSK3*β*, Snail1, E-cadherin, and hENT1 bands were determined. The levels of Snail1, E-cadherin, and hENT1 are reported relative to those of *β*-actin, and the levels P-GSK3*β* are reported relative to those of GSK3*β*. The values shown are the mean±standard error of the mean of six mice (**a** and **b**) from each group, and the mean±S.D. of three mice (**d**). The *P* value was calculated using two-way ANOVA. NS, not significant; *P<0.05; **P<0.01 (**a**, **b** and **d**)

Because zidovudine stimulated gemcitabine-induced cell death in PK1-GR and KLM1-GR cells *in vitro*, we sought to determine whether co-administration of zidovudine and gemcitabine inhibits gemcitabine-resistant tumor growth *in vivo* using a tumor xenograft model. Nude mice were xenografted with PK1-GR or PK1 cells. Gemcitabine was intraperitoneally administered once every 2 days, and zidovudine was orally administered once daily. Tumors induced by PK1-GR cells aggressively grew compared with those induced by PK1 cells in the gemcitabine-administered condition. As expected, co-administration of zidovudine and gemcitabine to mice with tumors induced by PK1-GR cells significantly reduced tumor volume (Figure 6a).

Our results indicated that co-treatment with zidovudine and gemcitabine is an effective new combination treatment for pancreatic cancer by targeting gemcitabine-resistant cells. To further confirm these results, we treated nude mice with PK1-cell xenografts by the intraperitoneal administration of gemcitabine once every 3 days and oral administration of zidovudine once daily. Administration of gemcitabine to mice with PK1 tumors reduced tumor volume at 4 weeks compared with non-treated tumors; however, the tumor volume started to increase at 2 weeks (Figure 6b). Co-administration of gemcitabine and zidovudine significantly reduced tumor volume compared with gemcitabine treatment alone at 4 weeks (Figure 6b). We performed immunoblotting analysis to determine whether the administration of gemcitabine induced the gemcitabine-resistant phenotype and the zidovudine effect on this phenotype in the xenografted tumors *in* *vivo*. After 4 weeks of gemcitabine administration, the expression levels of P-GSK3*β* and Snail1 were increased, and the expression level of hENT1 was significantly suppressed, suggesting that gemcitabine-treated xenograft tumors show the same gemcitabine-resistant phenotype as shown *in vitro* (Figures 6c and d). Moreover, co-administration of gemcitabine and zidovudine suppressed the expression of levels of P-GSK3*β* and Snail1 as well as increased the expression level of hENT1 compared with the administration of gemcitabine alone (Figures 6c and d). At week 1, the expression of these proteins did not change in the xenografted tumors (Figure 6c right panel). These results suggested that co-administration of gemcitabine and zidovudine to tumors dramatically suppresses tumor volume via inhibition to obtain the gemcitabine-resistant phenotype in pancreatic tumors. Together, the xenograft model data indicated that the gemcitabine and zidovudine co-treatment has potential as a new strategy for pancreatic cancer treatment.

## Discussion

Here, we identified zidovudine as a selective killer of gemcitabine-resistant pancreatic cancer cells by enhancing gemcitabine toxicity. We showed that gemcitabine-resistant cells have an EMT-like phenotype, and these cells have downregulated expression levels of hENT1 *in vitro* and *in vivo* via induction of Snail1 by the activated Akt-GSK3*β* pathway. Furthermore, zidovudine stimulates gemcitabine-induced cell death in gemcitabine-resistant cells by inhibiting the activated Akt-GSK3*β*-Snail1 pathway. More importantly, co-administration of gemcitabine and zidovudine effectively suppresses the growth of xenografted pancreatic tumors compared with gemcitabine alone by suppressing the gemcitabine-resistant phenotype.

This is the first report to show that zidovudine resensitizes gemcitabine-resistant cultured and xenografted pancreatic cancer cells to gemcitabine-induced cell death. An important aspect of this study was the use of gemcitabine-resistant cells, which were established by exposure to gemcitabine, for drug screening and the following analysis. These established cells were more resistant to high concentrations of gemcitabine (PK1 cells: IC_50_=0.9 *μ*M; PK1-GR cells: IC_50_>20 *μ*M) than other cultured gemcitabine-resistant cell lines.^32, 33^ Cultured gemcitabine-sensitive and -resistant cell lines have been mainly used for these types of screening in previous reports.^34, 35^ It may be difficult to identify drugs that enhance gemcitabine-induced cell death using highly gemcitabine-resistant pancreatic cancer cells. However, analyzing cells that have become resistant as a result of drug exposure has provided new insights into the mechanism of drug resistance and treatment methods for pancreatic cancer.^15, 36^ Similarly, our screening method found that zidovudine has high potential as an anti-cancer drug and is a useful biochemical tool to investigate the primary molecular mechanisms of gemcitabine resistance.

Accumulating evidence has strongly suggested that downregulation of hENT1 expression, a gemcitabine transporter, is involved in gemcitabine resistance in the clinic.^11, 13^ However, the molecular mechanism of the regulation of hENT1 expression is unknown. hENT1 is an essential component in cellular biology for the transport of nucleosides, and it is widely distributed and abundantly expressed in several tissues.^10^ The mechanisms of hENT1 expression regulation have not been completely elucidated, but reports have suggested that PPAR*α*, *γ* induces hENT1 expression and that NO signaling suppresses hENT1 expression.^37, 38^ We revealed that Akt-GSK3*β* signaling regulates hENT1 expression and that P-GSK3*β* suppresses hENT1 expression through a chemical biology method using zidovudine and a GSK3*β* inhibitor. Furthermore, we demonstrated that Snail1, a well-known EMT-inducing transcriptional factor, is involved in the downregulation of hENT1. The effects of zidovudine and Snail1 on hENT1 mRNA are weaker than those on hENT1 protein expression, suggesting that zidovudine-induced Snail1-dependent hENT1 expression is regulated by both transcriptional and nontranscriptional mechanisms. We also demonstrated that TGF-*β*, an EMT inducer, suppresses hENT1 expression, thus suggesting that the EMT phenotype is related to the regulation of hENT1 expression.

The mechanism by which zidovudine potentiates the cell death effects of gemcitabine may involve suppression of the Akt-GSK3*β* pathway. Akt signaling has been linked with chemoresistance.^39, 40, 41^ Thus, it is likely that inhibition of Akt by zidovudine sensitized gemcitabine-resistant cells to gemcitabine. These results are consistent with a previous report in which the Akt inhibitors LY294002 and wortmannin enhanced the gemcitabine effect in pancreatic cancer.^42^ Similarly, the Akt inhibitor MK-2206 has been shown to sensitize lung cancer cells to gemcitabine.^43^ Our results showed that zidovudine inhibited Akt phosphorylation in only gemcitabine-resistant cells, suggesting that zidovudine may specifically inhibit the activated pathway that phosphorylates Akt in PK1-GR cells. However, further studies are required to identify the primary target of zidovudine.

We also evaluated the anti-cancer effect of zidovudine and gemcitabine co-treatment *in vivo* and the phenotype of gemcitabine resistance using a tumor xenograft model. Single administration of gemcitabine to tumors induced by gemcitabine-sensitive cells suppressed growth until week 2. At week 4, the tumor volume then started to increase, and the tumor had a gemcitabine-resistant phenotype (EMT-like phenotype and downregulation of hENT1). This result indicated that gemcitabine treatment *in vivo* induced gemcitabine resistance, which strongly supported the *in vitro* results. An important aspect of the present study is the demonstration that co-administration of zidovudine and gemcitabine effectively inhibited the growth of tumors induced by pancreatic cancer cells in nude mice compared with gemcitabine alone, thereby suggesting that zidovudine may serve as an anti-cancer drug that targets gemcitabine-resistant tumors.

In conclusion, our data demonstrated that the Akt-GSK3*β*-Snail1→hENT1/EMT regulatory circuit represents a novel mechanism for establishing gemcitabine-resistant signaling. Moreover, we propose the zidovudine and gemcitabine co-treatment may be a novel therapeutic strategy for pancreatic cancer by targeting the gemcitabine-induced EMT-like phenotype and suppression of hENT1 expression.

## Materials and Methods

### Xenograft model

The Animal Research Committee of Kochi Medical School approved all experimental protocols and surgical procedures (Permit Number: H-00023). Each BALB/c nude mouse (Crea-Japan Inc., Tokyo, Japan; female; 5 weeks of age) was subcutaneously inoculated in the right and left hind footpads with 5 × 10^6^ PK1 or PK1-GR cells. For the PK1-GR cells, 2 days after inoculation, DMSO or gemcitabine (10 mg/kg) was intraperitoneally administered once every 2 days, and zidovudine (100 mg/kg) was dissolved in 1% methylcellulose and administered orally once daily. For the PK1 cells, 7 days after inoculation, DMSO or gemcitabine (10 mg/kg) was intraperitoneally administered once every 3 days, and zidovudine (100 mg/kg) was dissolved in 1% methylcellulose and administered orally once daily. Tumors were measured every 7 days, and their volumes were calculated using the following equation: mm^3^=[length (mm)] × [width (mm)]^2^/2).

### Cell lines and generation of stably transfected cell lines

The human pancreatic cancer cell lines, PK1 and KLM1, were maintained in DMEM supplemented with 10% FBS, 100 U/ml penicillin, and 100 *μ*g/ml streptomycin. PK1 and KLM1 cells were obtained from the RIKEN Cell Bank (Ibaraki, Japan).

Exposure of PK1 and KLM1 cells to increasing gemcitabine concentrations starting at 10 nM generated gemcitabine-resistant cells. As the cells adapted to the gemcitabine during culture for at least 2 weeks, the gemcitabine concentration was doubled. Finally, the cells were adapted and grew logarithmically with 1000 nM gemcitabine. The cell lines were named PK1-GR and KLM1-GR.

PK1-GR and KLM1-GR cells were transiently transfected using lipofection with plasmids that expressed hENT1.

### Immunoblotting analysis

Immunoblotting experiments were conducted as previously described.^44^ The antibodies used for immunoblotting were specific for the following proteins: P-ERK1/2 (Thr 202/Tyr 204), P-Smad3 (Ser 423/425), P-Akt (Ser 473), P-GSK3*β* (Ser 9), GSK3*β*, *β*-catenin, and Snail1 (Cell Signaling, Beverly, MA, USA); α-SMA and *β*-actin (Sigma, St. Louis, MO, USA); E-cadherin (Life Technologies, Carlsbad, CA, USA); and hENT1 (Santa Cruz, Dallas, TX, USA). Antibodies were diluted to 1 : 1000, except for anti-*β*-actin (1 : 10 000). Secondary antibodies were purchased from Promega (Madison, WI, USA; anti-rabbit and anti-mouse at 1 : 5000).

### Real-time quantitative PCR (qRT-PCR)

qRT-PCR and RT-PCR were conducted as previously described.^44^ To normalize the amount of total RNA present in each reaction, *β-actin* cDNA served as an internal standard. The following primers were used (name, forward primer and reverse primer): *α-SMA*, 5′-CATCATGCGTCTGGATCTGG-3′ and 5′-GGACAATCTCACGCTCAGCA-3′ *β-actin*, 5′-GGACTTCGAGCAAGAGATGG-3′ and 5′-AGCACTGTGTTGGCGTACAG-3′ *Snail1*, 5′-GCCTAGCGAGTGGTTCTTCT-3′ and 5′-TAGGGCTGCTGGAAGGTAAA-3′ *Slug*, 5′-GAGCATTTGCAGACAGGTCA-3′ and 5′-ACAGCAGCCAGATTCCTCAT-3′ *E-cadherin*, 5′-TGCCCAGAAAATGAAAAAGG-3′ and 5′-GTGTATGTGGCAATGCGTTC-3′ *hENT1*, 5′-ACGATGCCTGGTTCATCTTC-3′ and 5′-CCTCA;GCTGGCTTCACTTTC-3′ *dCK*, 5′-GCCACTCCAGAGACATGCTT-3′ and 5′-CTATGCAGGAGCCAGCTTTC-3′ *RRM1*, 5′-CATCCACATTGCTGAGCCTA-3′ and 5′-GATTAGCCGCTGGTCTTGTC-3′ and *RRM2*, 5′-CTGGCTCAAGAAACGAGGAC-3′ and 5′-GTTTGAACATCAGGCAAGCA-3′.

### Cell viability assays

Cell viability was determined using the 3-(4,5-di-methylthiazol-2-yl)-2,5-diphenyltetrazolium bromide (MTT) method.^45^ After treatment with indicated drugs, cells were incubated with the MTT solution (1 mg/ml) for 2 h. Isopropanol and HCl were added to final concentrations of 50% and 20 mM, respectively. The optical density at 570 nm was determined using a spectrophotometer with a reference wavelength of 630 nm.

### siRNA experiments

PK1 and KLM1 cells were transfected with a siRNA specific for Snail1 (5′-GCGAGCUGCAGGACUCUAA-3′),^46^ hENT1 (5′-GAUCGUGCUCAUUAAUUCA-3′)^47^ and Control (Santa Cruz) at final concentrations of 50 nM using the X-tremeGENE transfection reagent (Roche, Basel, Schweiz) according to the manufacturer's instructions.

### Cell invasion assay

The invasion assay was performed using BioCoat Matrigel invasion chamber system (Corning, Corning, NY, USA) according to the manufacturer's protocol with some modifications. The lower chamber of the Transwell was filled with culture; the cell suspension containing 1% FBS was applied to the Matrigel-coated upper chamber medium containing 10% FBS. The plate was incubated at 37 °C for 24 h. Cells were removed from the upper surface of the membrane and the lower surface of the membrane was stained for 10 min with 1% crystal violet in 25% methanol, rinsed with distilled water, and air dried. The crystal violet was then extracted with 0.1 M sodium citrate in 50% ethanol and the absorbance was measured at 570 nm.

### Cell migration assay

*In vitro* wound-healing assays were used to assess cell migration as previously described.^48^ Confluent PK1 or PK1-GR cells on a 24-well plate were used. Two linear wounds were scratched with a p200 pipette tip. The cell-free area was measured before and after 24 h of incubation (healing step) using the ImageJ software (Wayne Rasband, NIH).^49^

### Statistical analysis

Differences between mean values were evaluated using two-way ANOVA followed by Tukey's test. Differences were considered significant at *P*<0.05.

## Abbreviations

EMT: epithelial to mesenchymal transition
dCK: deoxycytidine kinase
hENT1: human equilibrative nucleoside transporter 1
RRM: ribonucleoside diphosphate
α-SMA: α-smooth muscle action
TGF: transforming growth factor
qRT-PCR: real-time quantitative PCR
MTT: 3-(4,5-di-methylthiazol-2-yl)-2,5-diphenyltetrazolium bromide
