# Supplementary material for: Zidovudine, an anti-viral drug, resensitizes gemcitabine-resistant pancreatic cancer cells to gemcitabine by inhibition of the Akt-GSK3β-Snail pathway
Source: Cell Death Dis. 2015 Jun 25;6(6):e1795–. doi: 10.1038/cddis.2015.172 (PMC4669843; doi:10.1038/cddis.2015.172)
Supplement: Supplementary Information [file cddis2015172x1.docx]

**Supplementary Figure S1.** (A) Zidovudine had the expected effect of resensitizing the cells to gemcitabine in screened compounds. PK1-GR cells were co-treated with gemcitabine (1 µM) and indicated compound for 72 h using the procedure described in Figure 1. Data are presented as the average of two independently performed experiments. (B) Zidobudine did not induce cell death in PK1-GR and KLM1-GR cells. PK1-GR and KLM1-GR cells were treated with zidovudine at indicated concentration for 72 h using the procedure described in Figure 1.

**Supplementary Figure S2.** Zidovudine upregulates hENT1 expression and suppresses the EMT-like phenotype via activation of GSK3β, and this induction is involved in the zidovudine-dependent stimulation of gemcitabine-induced cell death in KLM1-GR cells. (A) KLM1 or KLM1-GR cells were incubated with zidovudine (10 µM) for 48 h. Immunoblotting analysis was performed using the same procedure described in Figure 4. (B) hENT1 was transiently expressed in KLM1-GR cells. After 24 h, these cells were treated with gemcitabine (1 µM) for 48 h. Immunoblotting and MTT assays were performed using the same procedure described in Figure 4 (C) KLM1-GR cells were transfected with siControl or siSnail1 for 48 h. Left panel: Total RNA was extracted and subjected to qRT-PCR analysis using the indicated specific primer sets. Right panel: Whole-cell lysates were subjected to immunoblotting analysis with the indicated antibodies. (D) KLM1-GR cells were transiently transfected with siControl or siSnail1 and were treated with gemcitabine (1 µM) for 48 h. Cell viability was determined using an MTT assay. (E) KLM1 or KLM1-GR cells were incubated with zidovudine (10 µM) for 48 h. Immunoblotting analysis was performed using the same procedure described in Figure 4. (F) KLM1-GR cells were treated with gemcitabine and simultaneously co-cultured with zidovudine (10 µM) and/or BIO (10 µM) for 48 h. Immunoblotting and MTT assays were performed using the same procedure described in Figure 5. The *P* value was calculated using two-way ANOVA. **P*<0.05; ***P*<0.01 (B-D, F).

**Supplementary Figure S3.** The EMT inducer, TGF-β, suppresses hENT expression. PK1 cells were treated with TGF-β (100 ng/ml) for 48 h. Total RNA was extracted and subjected to qRT-PCR analysis using the indicated specific primer sets.
