# Supplementary figures and images for: Zidovudine, an anti-viral drug, resensitizes gemcitabine-resistant pancreatic cancer cells to gemcitabine by inhibition of the Akt-GSK3β-Snail pathway
Source: Cell Death Dis. 2015 Jun 25;6(6):e1795–. doi: 10.1038/cddis.2015.172 (PMC4669843; doi:10.1038/cddis.2015.172)

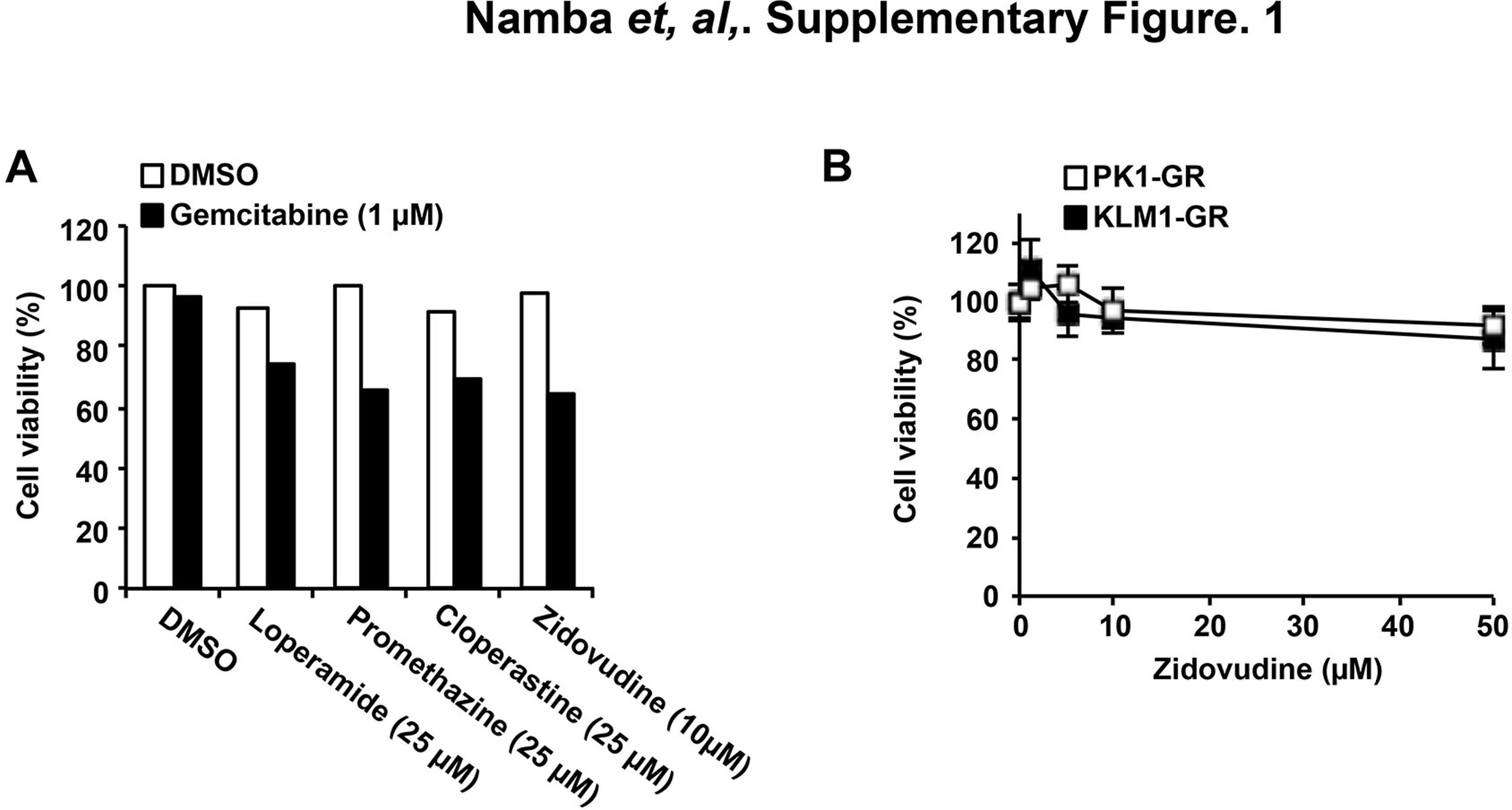

Supplement: Supplementary Figure 1 [file cddis2015172x2.tif]

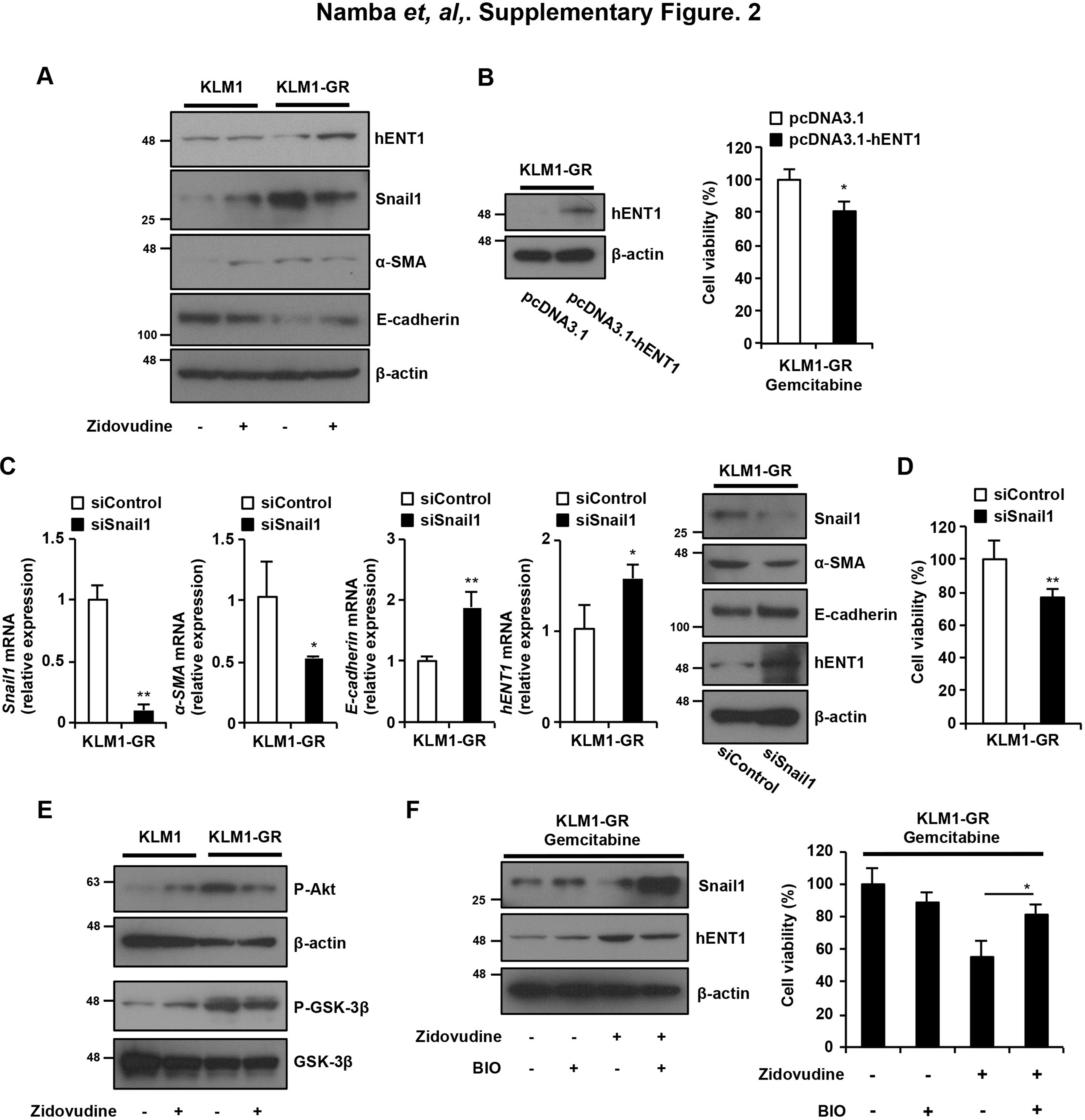

Supplement: Supplementary Figure 2 [file cddis2015172x3.tif]

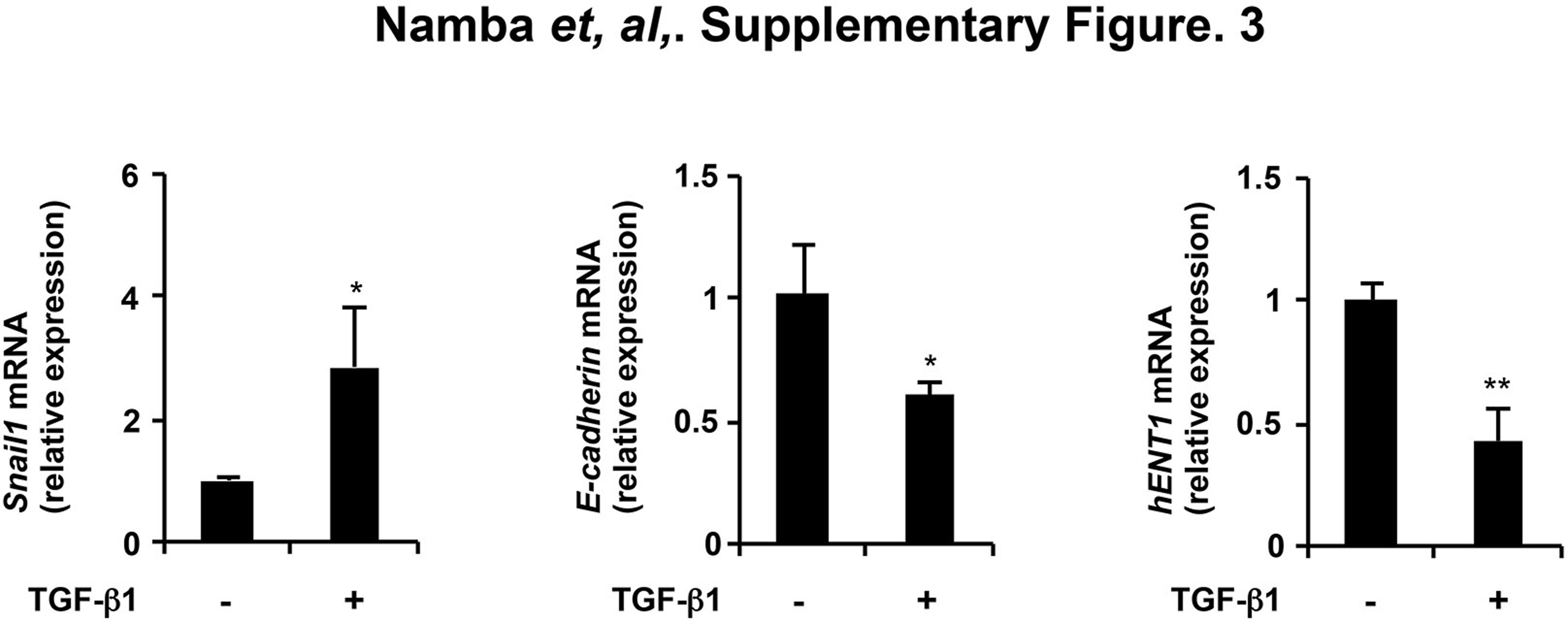

Supplement: Supplementary Figure 3 [file cddis2015172x4.tif]
